# Quantifying Bias in Hierarchical Category Systems

**DOI:** 10.1162/opmi_a_00121

**Published:** 2024-03-01

**Authors:** Katie Warburton, Charles Kemp, Yang Xu, Lea Frermann

**Affiliations:** School of Psychological Sciences, University of Melbourne, Melbourne, Australia; Department of Computer Science, University of Toronto, Toronto, Ontario, Canada; Cognitive Science Program, University of Toronto, Toronto, Ontario, Canada; School of Computing and Information Systems, University of Melbourne, Melbourne, Australia

**Keywords:** categorization, bias, western bias, gender bias, library classification systems

## Abstract

Categorization is ubiquitous in human cognition and society, and shapes how we perceive and understand the world. Because categories reflect the needs and perspectives of their creators, no category system is entirely objective, and inbuilt biases can have harmful social consequences. Here we propose methods for measuring biases in hierarchical systems of categories, a common form of category organization with multiple levels of abstraction. We illustrate these methods by quantifying the extent to which library classification systems are biased in favour of western concepts and male authors. We analyze a large library data set including more than 3 million books organized into thousands of categories, and find that categories related to religion show greater western bias than do categories related to literature or history, and that books written by men are distributed more broadly across library classification systems than are books written by women. We also find that the Dewey Decimal Classification shows a greater level of bias than does the Library of Congress Classification. Although we focus on library classification as a case study, our methods are general, and can be used to measure biases in both natural and institutional category systems across a range of domains.^1^

## INTRODUCTION

Categories inevitably reflect the needs, perspectives, and experiences of the people who create them (Bowker & Star, 2000). Consider, for example, Steinberg’s famous depiction of the *View of the World from 9th Avenue*^2^, which devotes half of the page to three New York City blocks but shows China, Russia, and Japan as tiny blobs on the horizon. A *View of the World from Tiananmen Square* would look rather different, and might include separate categories for Shānxī and Shǎnxī provinces while making no distinction between Washington State and Washington DC.

Although systems of categories are often subjective, the distinctions that they encode or fail to encode can have important consequences (Crawford, 2021). For example, Gould (1990) points out that the United States’ categorization of drugs as legal or illegal results in some addictive drugs being advertised on TV, and others carrying life sentences. The categorization of an animal or plant population as a distinct species as opposed to a variant of an existing species can affect conservation efforts and biodiversity research (Freeman & Pennell, 2021; Thomson et al., 2021). Finally, categories can also lead to harmful stereotypes, especially when coarse categories are used for members of out-groups in contrast to the finer-grained categories used for members of one’s in-groups (Park & Rothbart, 1982). Because category systems can encode and reinforce stereotypes, it is important to ensure that the biases encoded by these systems are acknowledged as such instead of treated as ground truth.

Social, developmental and cognitive psychologists have previously explored how categories (e.g., racial and gender categories) arise and how these categories influence behaviour (Brewer, 2007; Misch et al., 2022; Timeo et al., 2017; Waxman, 2021). Existing work highlights two key connections between categorization and bias. First, people tend to have positive attitudes towards in-group categories and negative attitudes towards out-group categories, and methods such as the Implicit Association Test (Greenwald et al., 1998; Schimmack, 2021) attempt to measure these attitudes. Second, categories can bias the way in which people perceive individual members of in-groups and out-groups. For example, there is a tendency to perceive members of one’s out-group as being more similar to one another than are members of one’s ingroup (Judd & Park, 1988; Mackie & Worth, 1989; Park & Rothbart, 1982; Rubin & Badea, 2012). The link between categorization and biased perception has been explored more generally (Dubova & Goldstone, 2021; Goldstone et al., 2001), and the tendency to overestimate both within-category similarity and between-category distinctiveness is sometimes referred to as categorization bias (Ashby & Zeithamova, 2022) or categorical bias (Ester et al., 2020). Here we focus on a third connection between categorization and bias, and consider ways in which the structure of a category system (i.e., the extensions of the categories that it includes) can reflect bias. A canonical example is that a category system may include fine-grained categories in areas that are deemed valuable or important, and coarser categories in areas deemed less worthy of attention.

The term “bias” has been used to refer to distinct concepts in the literature. For example, “inductive bias” refers to constraints or expectations that guide learning (Griffiths et al., 2010; Markman, 1989), and is not directly relevant to our study. We define bias as preferential treatment for one group (e.g., western individuals) over another (e.g., non-western individuals). As we discuss later, this bias can either reflect external biases that have shaped the items to be categorized, or can be internal to a category system and imposed on the items by this system. To study this notion of bias we develop methods to measure how different groups are represented in a category system. Establishing that a system is biased requires us to demonstrate that the system departs from an unbiased alternative, and it is not always clear how an unbiased system should weigh different groups. We therefore begin with the simplifying assumption that an unbiased system should give roughly equal weight to each group, but return to this assumption later and discuss the extent to which it is appropriate for the specific oppositions that we consider (western vs non-western, and male vs female and non-binary).

Much of the previous work on bias in categorization has focused on biases in individual categories or in flat category systems. However, items can be categorized at multiple levels of abstraction (Mervis & Rosch, 1981) and natural categories are often organized into conceptual hierarchies. For example “flower” is a subcategory of “plant” which is a subcategory of “living things”. In addition, many formalized systems of categories such as biological taxonomies, medical ontologies, and library classification systems have hierarchical structures. Here we consider several kinds of biases that can occur in hierarchical category systems. Some of these biases have counterparts in flat systems: for instance, cat lovers could have more fine-grained category divisions for cat breeds than they do for dog breeds. Other biases, however, are distinctive to hierarchical systems. For example, Loehrlein (2012) demonstrated that people are biased towards concepts located near the top of a hierarchical system such that these concepts are perceived as being more important than those at the bottom.

Our methods are general and could potentially be used to measure bias in any hierarchical category system. Laboratory methods commonly used to elicit hierarchical category systems include successive pile sorting and hierarchical clustering based on judgments of similarity (Medin et al., 1997, 2006), and our approach could be applied to hierarchies generated by any such method. Another example is WordNet (Miller, 1994), a lexical database that organizes nouns and verbs into hierarchies. WordNet is both an influential theory of human lexical memory and a resource used to develop and test many other theoretical contributions in cognitive science, and understanding bias in WordNet is therefore important. WordNet aims for very broad coverage of the lexicon, and a separate research tradition aims to document folk taxonomies of specific semantic domains including plants, animals, artifacts, diseases, and soils (Holman, 2005). Our methods could be used to identify areas given more and less weight by these taxonomies—for example, we could measure the extent to which an animal taxonomy privileges domesticated animals ahead of wild animals, and compare the strength of this bias across cultures. Our approach may therefore be broadly useful as a tool for studying the way in which people’s category systems are influenced by cultural and individual biases, and for exploring the idea that category systems are not exclusively shaped by intrinsic properties of the things that they categorize, but are instead heavily influenced by human needs and values.

Although our approach has many possible applications, here we take library classification as a case study and apply our methods to the Library of Congress Classification (LCC) and the Dewey Decimal Classification (DDC). These library classification systems are large-scale, hierarchical examples of human categorization that are directly accessible and much more amenable to computational analysis than the category systems that all of us carry around in our heads. Focusing on library classification also allows us to connect our approach with a large body of existing work in the library and information sciences devoted to uncovering and mitigating bias in the LCC (Angell & Price, 2012; Howard & Knowlton, 2018; Intner & Futas, 1996; Kam, 2007; Rogers, 1993), the DDC (Higgins, 2016; Kua, 2008; Olson & Ward, 1997; Westenberg, 2022), or both (Mai, 2010; Zins & Santos, 2011). Category systems, especially more formal systems like library classifications, are often perceived as neutral or objective, making it all the more important to develop methods that enable us to quantify and thus acknowledge and address the biases that may be implicit in these systems. As such, studying formal systems like these is valuable in its own right and can also contribute to a better understanding of categorization in general (Glushko et al., 2008).

Previous work on bias in the LCC and DDC has documented that the language used to label categories, and the location of topics and books in the classification schemes can encode harmful bias. For example, in the LCC unglossed religious terms like “God,” and “devotional literature” refer to these concepts only in the context of Christianity (Knowlton, 2005). Similarly, subject headings such as “engineers” that have subheadings such as “women engineers” but not “male engineers” assume men as the default (Rogers, 1993). In general, the LCC and DDC systems have been found to be biased and unsystematic in their coverage of non-western religions and racial groups (Westenberg, 2022; Zins & Santos, 2011) and both systems are biased in their categorization of non-western languages and literatures (Higgins, 2016; Howard & Knowlton, 2018; Kua, 2008). In addition, both systems struggle to represent topics related to women and women’s studies, and these topics are often restricted to limited sets of categories that are scattered across the classification scheme (Intner & Futas, 1996; Olson & Ward, 1997). We thus apply our methods to two case studies of bias. The first case study measures western bias, or bias in favour of western culture, in the categories of the LCC and DDC. The second measures gender bias, and compares the representation of books written by women to books written by men in both systems.

Our work systematically quantifies the extent of bias within the two library classification systems that we consider. For institutional category systems such as these, quantifying bias is important because a quantitative measure can be used to identify the parts of a system that show the strongest bias and are therefore most important to consider when proposing future improvements to the system. Quantifying bias in category systems is also important because categories can influence perception and behaviour (Goldstone et al., 2001; Loehrlein, 2012) and it is therefore important to understand the extent to which a system might bias its users’ understanding of the items that it categorizes. A third benefit of a quantitative approach is that it allows for the comparison of bias across two or more related classification systems, and we illustrate by comparing the LCC and the DDC. Finally, our quantitative approach can be applied at a relatively large scale, and therefore allows us to analyze many more items and categories than a single researcher would be able to process on their own.

We begin in the next section by providing background on the LCC and DDC. Next, we distinguish between category bias, or bias reflected in the internal nodes of a hierarchical system, and item bias, or bias reflected in the distribution of items to be classified. We then focus on western category bias and item gender bias in particular and present quantitative analyses that measure and compare the extent to which these biases are present in the LCC and DDC. We finish by demonstrating how our approach generalizes to other category systems through a case study of category bias in WordNet. We discuss limitations and future directions, including a broader perspective of how our methods could be used to quantify cultural and individual differences in diverse category systems.

## LIBRARY CLASSIFICATION SYSTEMS

**Figure 1.**
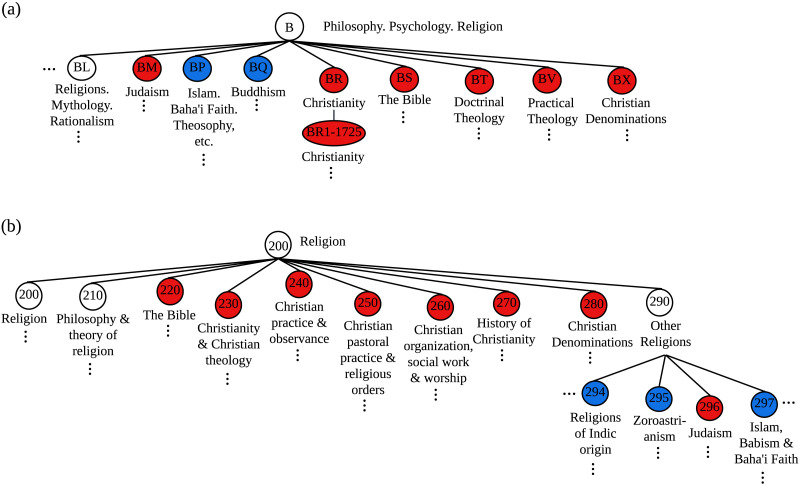
An example of how Religion is categorized in (A) the LCC and (B) the DDC. Red categories are tagged as western and blue as non-western. White categories cannot be tagged as solely western or non-western. For each library classification system, only a subset of the relevant categories is shown.

The LCC and DDC are both hierarchical systems that contain a set of main classes, each corresponding to a different discipline. These main classes are recursively subdivided into increasingly more specific subcategories that classify smaller and smaller subsets of the literature. In the LCC there are 21 main classes and classification numbers are alphanumeric. There is no formal limit on the number of subcategories a category can have. Figure 1A illustrates this system with the classification of religious literature. The DDC has 10 main classes and classification numbers are entirely numeric. Each category can have a maximum of 10 children. Figure 1B illustrates the classification of religious literature in the DDC. The category hierarchy is represented by the position of the digit that differentiates a category. The DDC has stronger structural constraints than the LCC as it enforces the strict upper limit on the number of subcategories (Svenonius, 2000). As a result, the LCC tends to be flatter and the DDC deeper.

### Library Classifications as Trees

We used a tree structure representation to capture the hierarchical structure of the LCC and the DDC. Every node in the tree represents a different category in the classification, storing a category’s name, label, and the books it classifies. The category label indicates the range of classification numbers that fall under it. The branches between a parent and its children represent the hierarchical relationship between a category and its subdivisions.

We used the OhioLINK Circulation Data^3^, a large publicly available data set of books and their circulation, to represent the books in our analysis of bias in library classification systems. OhioLINK contains 6.78 million MARC bibliographic records^4^ for books and manuscripts in the Ohio academic libraries (OhioLINK Collection Building Task Force et al., 2011). These bibliographic records include the LCC and DDC classification assigned to a book. Only books that had both an LCC and a DDC classification were kept resulting in 3.32 million books. These books were placed into the DDC and LCC tree structures using their relevant classification numbers. For each book, we found the most specific category associated with its classification number, and then recursively added it to each parent category until the top of the tree was reached. This ensured that each parent category contained all the books of its subcategories. For each book we stored its author and circulation statistics. See Appendix B for more details.

## DEFINING BIAS

As suggested earlier, bias can be defined as a preference for one group ahead of another. This definition can be applied separately to the categories or internal nodes that lie within a hierarchical system and to the items that are found at its leaves, and we refer to these two cases as “category bias” and “item bias” respectively. For us, the items are books, but in general an “item” could be any entity (e.g., a person, thing, concept, or action) that a classification system categorizes.

### Category Bias

Assume that blue and red represent distinct but comparable labels that can be applied to the internal nodes of a hierarchical classification system. For example, in Figure 1 red categories are related to western topics and blue categories are related to non-western topics. Category bias occurs when the system gives preferential treatment to one group of nodes (e.g., red nodes) ahead of the other. We assume for now that an unbiased system treats red and blue categories identically.

**Figure 2.**
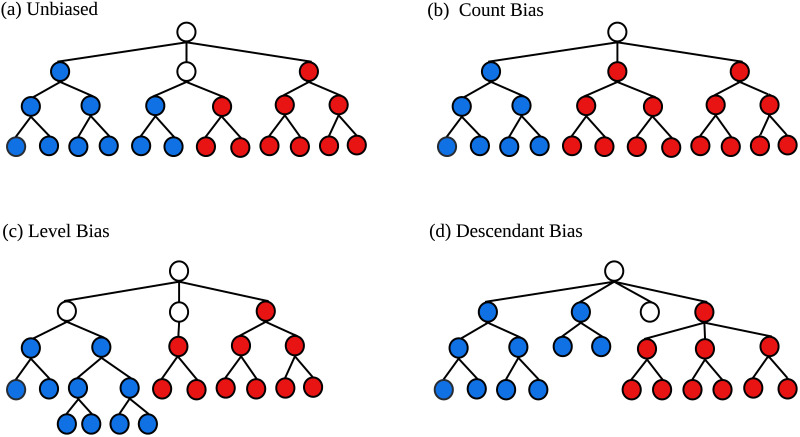
An illustration of (A) an unbiased classification, (B) a classification with count bias, (C) a classification with level bias, and (D) a classification with descendant bias. The three category biases demonstrate bias against blue categories and in favour of red categories. White categories cannot be categorized as either red or blue.

Three kinds of category biases are illustrated in Figure 2: count bias, level bias, and descendant bias. Category count bias (Figure 2B) occurs when there are more red than blue categories in a classification scheme. Thus, more classification space is devoted to red categories. Category level bias (Figure 2C) occurs when red starting categories occur higher in a classification structure than blue starting categories. A *starting category* (or starting node) is the first category in a classification sub-tree that can be labelled as red or blue. Starting categories that are higher in the classification scheme are conceptualized as more general or important than those that are deeper. Finally, descendant bias (Figure 2D) occurs when red starting categories have more descendants than blue starting categories on average. In other words, red categories are privileged by having more fine-grained category divisions.

The three biases in Figure 2 may often be correlated in practice—for example, if there are more red categories (category count bias) it is likely that red starting categories will have more descendants (descendant bias). The biases, however, are conceptually distinct and can be separated in principle. For example, Figure 2D shows that even when node counts are held constant for red and blue it is possible to observe level bias (in favour of blue) and descendant bias (in favour of red). We therefore propose that considering the three biases individually is worthwhile as they highlight different aspects of category bias.

### Item Bias

**Figure 3.**
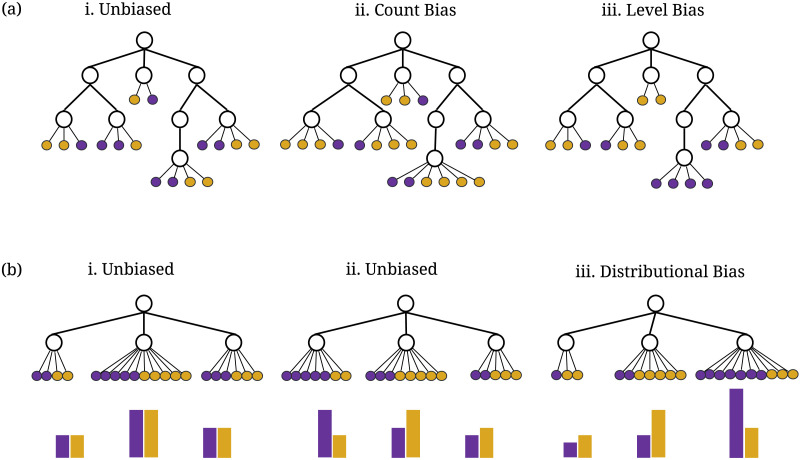
An illustration of (A) item count bias and level bias, and (B) distributional bias. In (A) there is (i) a classification with no level or count bias; (ii) a classification with count bias; and (iii) a classification with level bias. In (B) there are (i, ii) two examples of categorizations without distributional bias, and (iii) an example of distributional bias. The shapes of the distributions of purple and gold items across the three lowest categories in the tree are shown with bar graphs. All 3 item biases demonstrate bias against purple items and in favour of gold items.

Instead of assigning the internal nodes of a hierarchical system to groups, assume now that purple and gold represent distinct but comparable labels that can be applied to a set of items. For example, gold items could be books written by men and purple items could be books written by women and nonbinary people. Figure 3 shows several examples in which the items are shown as small circles at the leaves of a classification hierarchy. *Item bias* occurs when the system gives preferential treatment to one group of items (e.g., gold items) ahead of the other. As before, we assume that an unbiased system would give equal treatment to gold and purple items.

Three kinds of item biases are illustrated in Figure 3: count bias, level bias, and distributional bias. Item count bias (Figure 3A.ii) occurs when there are more gold than purple items classified by a system. Item level bias (Figure 3A.iii) is similar to category level bias, and occurs when gold items tend to be found higher in the classification tree than gold items. Finally, distributional bias (Figure 3B.iii) occurs when gold items are distributed more broadly across the classification system than are purple items. In other words, purple items are more restricted to a limited part of the classification scheme than are gold items.

Distributional bias can be diagnosed by comparing the shape of the distributions of gold items to the shapes of the distributions of purple items. In Figure 3B.iii, the distribution of the gold items across the three categories at the lower level of the hierarchy is relatively flat, but the purple distribution is concentrated on the third of the three categories. In contrast, Figures 3B.i and 3B.ii show distributions of purple and gold authors that do not suffer from distributional bias. In Figure 3B.i the shape of the distribution of purple authors is identical to the shape of the distribution of gold authors. In Figure 3B.ii, although the distributions are not identical, they are shuffled versions of one another and therefore equally as flat.

**Figure 4.**
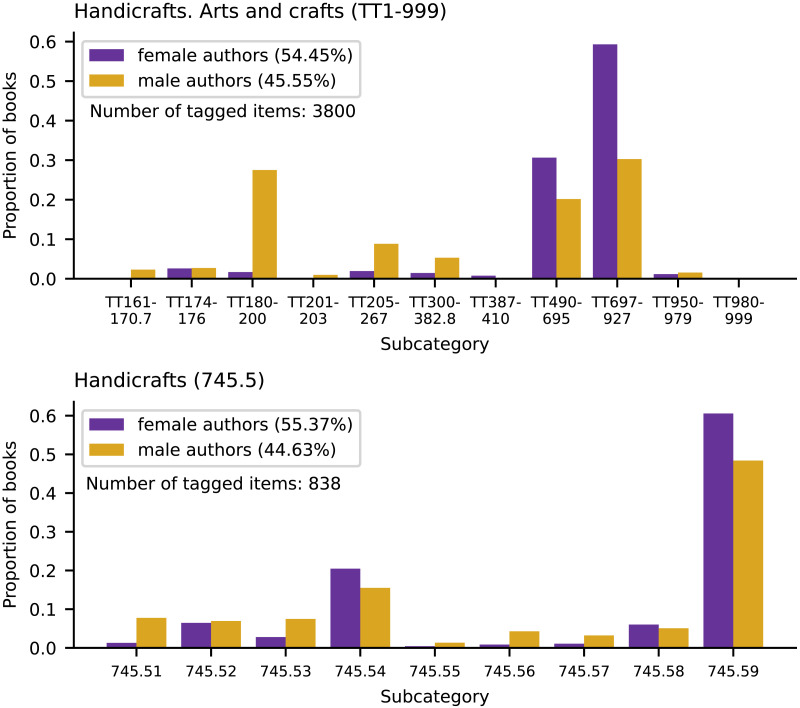
An example of the distributions of male and female authors in the category “Handicrafts. Arts and Crafts” in the LCC (top) and “Handicrafts” in the DDC (bottom). Number of tagged items is the number of books that have an author with a known gender in the dataset. See Appendix A for the full set of subcategory names.

Figure 4 shows what distributional bias can look like in library classification systems. In the LCC, the books by men are more evenly spread across the subcategories of “Handicrafts. Arts and crafts,” than are the books by women. The books by women are predominately restricted to two subcategories, “Home arts. Homecrafts” (TT697-927) and “Clothing manufacture. Dressmaking. Tailoring” (TT490-695). In the DDC’s equivalent category, “Handicrafts,” the difference between the shape of the distribution of books by men and books by women does not appear to be as big.

Item biases can arise for at least two different reasons. They can be attributed to how the classification system distributes items, to external social biases found in the set of items to be classified, or to both. We therefore distinguish between two kinds of item biases: external biases and internal biases. External biases are biases found in the set of items to be classified. These biases are the result of broader social forces and are not imposed by the classification system itself. Internal biases are biases in the ways items are assigned to categories by the classification system. These biases are imposed on the items by the classification system itself.

In the context of gender bias in library classification systems, item count bias is a clear example of external bias as it comes from unequal numbers of purple and gold authors in the set of items to be classified. This bias could arise because society provides more opportunities for men to write books than women, or because libraries are more likely to acquire books written by men (Quinn, 2012), or both. How to characterize distributional bias and level bias is less clear. For example, in the case of distributional bias in a library classification system, it might be that purple and gold authors write about equally diverse sets of topics, but that the interests of purple authors are given limited space in the classification system. Thus it could be that the distributional bias is an example of an internal bias. It could also be that the topics addressed by purple authors are genuinely less diverse than the topics addressed by gold authors because of social pressures that encourage purple authors to specialize in a limited set of areas. Thus the distributional bias could also be an example of an external bias. Similarly, level bias against purple items could be the result of the classification system placing topics of interest to purple authors lower in the tree (internal bias), or social pressures pushing purple authors to write in smaller, more niche categories (external bias). Although the origins of level bias and distributional bias may not be clear, both biases are worth investigating as they can provide insight into how different groups are represented in a classification scheme, regardless of whether this difference in representation is imposed by the system itself or the result of external forces.

## STUDY 1: WESTERN BIAS

One of the most well-documented biases in the LCC and the DDC is western bias, or bias in favour of western culture. In this study we use our definitions of category bias to quantify the degree of western bias in both systems.

### Methods

To study western category bias, we selected main classes in the classification trees that related to history, religion, language, and literature as these categories tended to have category names that could be identified as western or non-western. Other topics such as philosophy, although having the potential to exhibit western biases, did not have categories that could clearly be identified as western or non-western. For the LCC, we used main classes B, D, E, F, and P which are named “Psychology, Philosophy, Religion,” “World History,” “History of the Americas,” “History of the Americas (local)”, and “Language & Literature” respectively. For main class B we only used the categories that classified religious literature. For the DDC we used main classes 2, 9, 4, and 8 which are named “Religion,” “History,” “Language”, and “Literature.” We combined Language and Literature into a single topic because in the LCC many of the categories in main class P did not allow for easy separation of the two. Similarly, we grouped together all main classes related to history. We thus investigated western bias across three topics: religion, history, and language & literature.

We manually tagged the categories selected for each topic as western or non-western, drawing on distinctions that have been previously suggested in the literature.^5^ Still, the tagging process is inevitably subjective, and in cases where a label of western or non-western was unclear, we left the category untagged, aiming for precision over recall. This somewhat limits the results, as there might be cases where a country or language, etc. falls into a category with a clear label in the LCC but not the DDC or vice versa.

The classes related to history tended to be divided into categories based on geographical and political divisions such as country or continent. We therefore used a list of western countries that were defined based on a cultural definition of “western” as opposed to a political, economic, or geographical definition (de Espinosa, 2017; Hall, 2018; Trubetskoy, 2017). For example, Australia tends to be considered a western country despite not being geographically in the western hemisphere. 68 countries, about 35% of the world’s countries, were included in the list of western countries and we assumed that countries left off the list were non-western. For each history-focused main class, we considered all categories associated with a country and tagged them as western or non-western based on the list. The tagged category became a starting category. If a category represented a group of countries (i.e., a category for a continent or a region) and all the categories beneath it shared the same tag, then that broader category became the starting category and inherited the tag. Similarly, every category under a starting category inherited the starting category’s tag.

In the language and literature-related classes, some categories were related to regional divisions like the history-focused classes so we based our tagging on the list of western countries used previously. Examples of these categories include “German literature” and “Languages and literature of Eastern Asia, Africa, Oceania.” Some categories were related to language families so we considered where these languages or language groups originated from to make the tagging choice. “Romance languages” is one example. The main deviation from the tagging method used for history was how we tagged Indigenous languages and literature from North America, South America, and Oceania. Consistent with our cultural definition of the western concept (Hall, 2018), we tagged them as non-western even if they originated from a country or region that is listed as western.

Finally, for the main classes covering religion, we mostly tagged Abrahamic religions as western and other religions as non-western. The few exceptions included tagging Scientology as western and Islam as non-western. Islam is an Abrahamic religion, but we made the conservative decision to tag Islam as non-western, because the opposite decision would probably only increase any western bias that we might find. The categories Doctrinal Theology and Practical Theology were tagged as western because they have only been used to classify literature on Christianity (Zins & Santos, 2011).

### Results

In total there were 3009 categories on the topics of religion, language & literature, and history in the LCC, and 13,536 in the DDC.^6^ Based on the tagging method, 86.3% of categories could be tagged as either western or non-western in the LCC, and 91.4% in the DDC. We refer to tagged categories as “nodes” to be consistent with our use of a tree representation.

#### Category Count Bias.

To compute category count bias we compared the percentage of nodes tagged as western to the percentage of nodes tagged as non-western in both classification systems. In the LCC 2598 nodes were tagged (62.24% western). In the DDC there were 12,372 tagged nodes (69.35% western).

**Figure 5.**
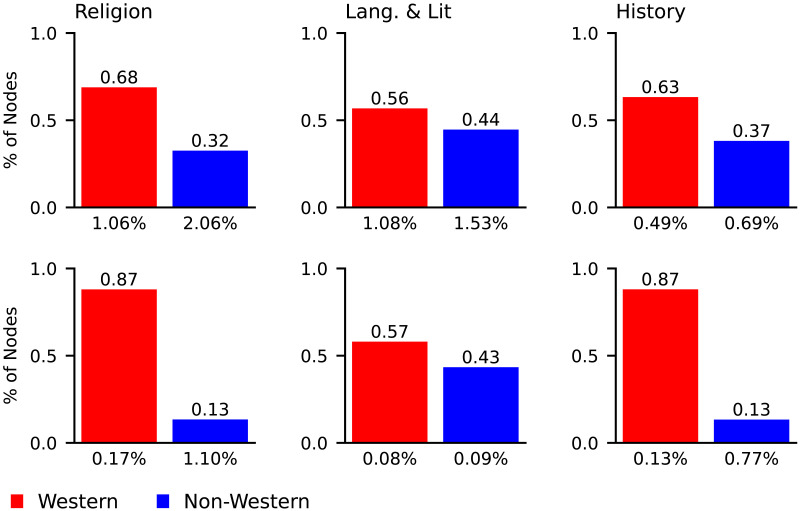
Category count bias results for religion, language & literature, and history in the LCC (top) and the DDC (bottom). The bars are labelled on the top with the proportion of nodes they represent. The labels below the *x* axis are the mean % of books per node.

Figure 5 shows analogous results for each of the three individual topics. For each topic, there is a higher percentage of nodes tagged as western than non-western. In the LCC, religion has the highest percentage of western nodes. In the DDC, history and religion had percentages that were almost equally high. For all topics, the DDC had a higher percentage of western nodes than the LCC. To test the statistical significance of this result we randomly assigned all nodes a western or non-western label with equal probability. We repeated the process 10,000 times, using the proportion of the times the absolute difference between western and non-western node counts was greater than or equal to the observed absolute difference as the *p* value. For all topics in the DDC, and religion and history in the LCC, *p* < 0.001. For language & literature in the LCC, *p* = 0.003. All category count biases were therefore statistically significant.

We have conservatively assumed that an unbiased system has an equal number of western and non-western nodes, but this assumption could be adjusted using statistics such as population sizes or the percentage of western countries. If anything, these statistics tend to suggest that an unbiased system should devote more space to non-western than to western nodes. For example, Africa and Asia accounted for 75% of the world’s population in 2022 (United Nations, DESA, Population Division, 2022). Western category count bias is substantial relative to a conservative 50–50 baseline, and would be even stronger relative to a a baseline favouring non-western nodes.

Library classification systems follow the principle of literary warrant, which means that their structures are derived from and justified by the body of literature that they classify (Svenonius, 2000). Based on this principle, it could be argued that there are more western categories because there are more western books that need to be classified. To test this idea, we calculated the mean rate of books per western node and non-western node in each system. These rates are reported as labels below the *x* axis of Figure 5. An unbiased system might be expected to have relatively equal rates of books per node. We found that language & literature in the DDC and history in the LCC have relatively equal rates of books per node for western and non-western nodes. Otherwise, there tend to be more books per non-western node than per western node. The difference in rates is most pronounced for religion (0.17% vs. 1.10%) and history (0.13% vs. 0.77%) in the DDC. These findings suggest that in some cases, especially in the DDC, the higher western category count cannot entirely be accounted for by literary warrant.

Similarly, it could be argued that there are more western nodes because western books are in higher demand than non-western books. To explore this idea we compared the circulation of books classified in western nodes to those in non-western nodes. Circulation statistics were drawn from the OhioLINK circulation data, and for each book we extracted three pieces of information: (i) whether the book was in circulation (i.e., available for borrowing) in 2007, (ii) whether the book was borrowed in 2007, and (iii) how often the book was borrowed in 2007. Mean values of all three variables are shown in Table 1. Across all topics and for both classification systems, a larger percentage of books classified under non-western nodes are in circulation than books classified under western nodes. For religion, a larger percentage of circulating non-western books were taken out than circulating western books in 2007 for both the LCC and DDC. In addition, among all religion books that were taken out, non-western books had a higher mean rate of circulation. The opposite was true for language & literature where a larger percentage of circulating western books were taken out and western books had a higher mean rate of circulation. For history, these statistics varied slightly but were relatively similar for western and non-western books. Overall, the circulation statistics do not seem to justify the large discrepancy between western and non-western node counts.

**Table 1.** Summary of circulation statistics for the LCC and DDC overall (O), and for topics religion (R), language & literature (LL), and history (H). Circulation is abbreviated to *circ*.

		**% books in circ.**		**% books taken out (2007)**		**Rate of circ. (2007)**	
		W	NW	W	NW	W	NW
**O**	**LCC**	0.92	0.97	0.27	0.27	2.78	2.62
	**DDC**	0.92	0.97	0.25	0.27	2.71	2.70
**R**	**LCC**	0.93	0.98	0.28	0.44	2.69	3.13
	**DDC**	0.93	0.98	0.28	0.44	2.65	3.12
**LL**	**LCC**	0.92	0.97	0.25	0.19	2.78	2.05
	**DDC**	0.92	0.97	0.23	0.18	2.72	2.08
**H**	**LCC**	0.92	0.97	0.28	0.28	2.86	2.68
	**DDC**	0.93	0.98	0.29	0.31	2.79	2.89

#### Level Bias.

To measure level bias we first compared the mean depth of western starting nodes (starting categories) to the mean depth of non-western starting nodes. If a group of nodes has a greater mean starting depth then this implies that, on average, the nodes occur deeper in the tree than a group with a lesser mean starting depth. The LCC has a mean western starting node depth of 2.28 and a mean non-western starting node depth of 3.35. In the DDC these depths are 2.77 and 3.57.

**Figure 6.**
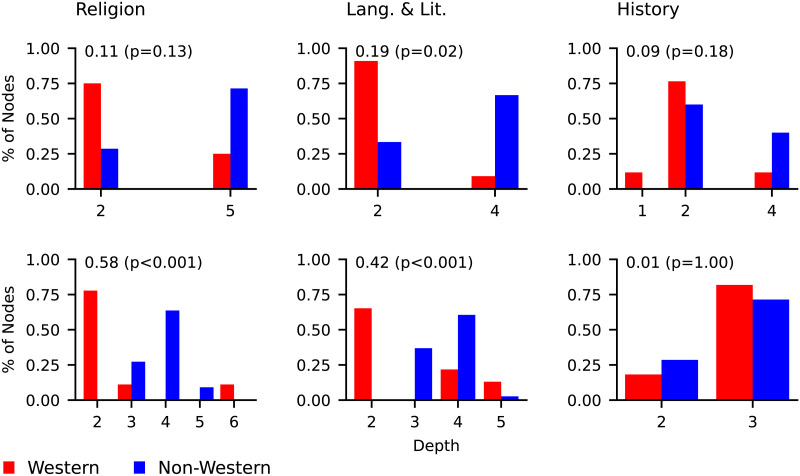
Level bias statistics for the topics religion, language & literature, and history in the LCC (top) and the DDC (bottom). Each plot is annotated with the JSD between the western and non-western distributions and the significance of the JSD in brackets. Significance was calculated with a permutation test.

For each topic, Figure 6 shows the distributions of starting nodes over classification tree depths. To quantify the difference in distributions over western and non-western starting depths, we computed the Jensen Shannon Divergence (JSD) between these distributions. To test the statistical significance of the results we performed permutation tests. For each topic, the depth labels were shuffled among the western and non-western nodes to create randomized depth distributions. This shuffling was carried out 10,000 times and the proportion of times the JSD between the randomized western and non-western depth distributions was greater than or equal to the actual JSD was used as the *p* value.

For all topics in the LCC, the mean depth of non-western starting nodes is greater than the mean depth of western starting nodes. The same finding applies to religion and to language & literature in the DDC. The one exception is history in the DDC, where the mean western depth is slightly greater than the mean non-western depth. For the two systems, the greatest divergences between western and non-western depth distributions occur for language & literature (LCC) and religion (DDC). In both classification systems, the divergence for history is not statistically significant (*p* = 0.18 and *p* = 1.00 in the LCC and DDC respectively). In the LCC, the divergence statistic was significant only for language & literature (*p* = 0.02), and for the DDC both religion (*p* < 0.001) and language & literature (*p* < 0.001) produced significant results. Religion did not demonstrate significant level bias in the LCC (*p* = 0.13).

The depths for the LCC and the DDC are not directly comparable, because the DDC tends to have a larger set of starting node depths than the LCC (e.g., the starting nodes for religion are spread across 5 different tree depths in the DDC versus just 2 in the LCC). We therefore computed an alternative measure of level bias that uses the probability that a randomly selected non-western starting node is deeper in a classification than a randomly selected western starting node. A western and a non-western starting node were randomly sampled 10,000 times. The depths of the two nodes were compared to determine the number of times the non-western one was deeper than the western one and vice versa (ties were ignored). The resulting statistic measured the probability that a non-western starting node would be deeper in a tree than a western starting node, given they were not of the same depth. The results are shown in Table 2. For every topic except history in the DDC, it is more likely that a randomly selected non-western starting node is deeper in the tree than a randomly selected western node. Western nodes for history in the DDC have a higher chance of starting deeper in the tree. Based on this statistic, the LCC displays a stronger level bias than does the DDC. To test for significance we performed a permutation test by randomly shuffling the depths among the western and non-western nodes and recalculating the probability that a non-western node was deeper in the tree. This was repeated 10,000 times and the proportion of times the absolute value of the difference between 50% and the recalculated probability was greater than the actual difference was used as the *p* values. The results are in Table 2. The significance by topic mirrored the significance of the initial divergence statistic for level bias.

**Table 2.** The probability that a randomly selected non-western starting node (NW) is deeper in the classification than a randomly selected western starting node (W), given the two nodes are not equal.

	**Prob. NW depth > W depth**		***p* value**	
	**LCC**	**DDC**	**LCC**	**DDC**
Overall	0.87	0.82	< 0.001	< 0.001
Religion	0.88	0.89	0.11	0.01
Lang. & Lit.	0.95	0.76	0.01	0.01
History	0.86	0.36	0.10	0.72

#### Descendant Bias.

We measure descendant bias by comparing the mean number of descendants per western starting node to the mean number of descendants per non-western starting node. We also recorded the number of starting nodes and the mean percentage of books per starting node. All statistics were computed for the LCC and DDC overall, as well as separately for religion, language & literature, and history. The results are shown in Table 3. To test for significance, the western and non-western tags were randomly shuffled among the starting nodes and the absolute difference in western and non-western descendant means was recomputed. This process was repeated 10,000 times, keeping track of the number of times the recomputed difference in means was greater than the absolute value of the observed difference in means.

**Table 3.** Summary of descendant bias for the LCC and DDC overall (O), and for topics religion (R), language & literature (LL), and history (H). Descendants is abbreviated to *desc*.

		**# of Start Nodes**		**% of Books per Start Node**		**Avg. # of Descendants per Start Node**	
		W	NW	W	NW	W	NW
**O**	**LCC**	36	26	2.8	3.8	43.9	36.7
	**DDC**	43	56	2.3	1.8	198.5	66.7
**R**	**LCC**	8	7	12.5	14.3	44.5	23.3
	**DDC**	9	11	11.1	9.1	196.7	22.5
**LL**	**LCC**	11	9	9.1	11.1	29.2	27.9
	**DDC**	23	38	4.3	2.6	183.7	82.2
**H**	**LCC**	17	10	5.9	10.0	53.2	54.1
	**DDC**	11	7	9.1	14.3	231.0	51.9

There is evidence of descendant bias in the DDC but not in the LCC. In the LCC, the difference in western and non-western descendant means was minimal and not statistically significant (*p* = 0.44 for religion, 0.92 for language & literature, and 0.97 for history). In contrast, all topics in the DDC had, on average, at least double the number of descendants per western starting node than non-western starting node. Descendant bias was statistically significant for the topics language & literature (*p* = 0.02) and religion (*p* < 0.001) but not for history (*p* = 0.62). Although history had the largest absolute difference in means, its lack of significance can be attributed to the fact that it has only 18 starting nodes and one of them (“History of the North Americas”) has a descendant count that is much larger than the other descendant counts.

The LCC has more western than non-western starting nodes. The DDC exhibits the opposite trend except in the case of history. The fact that there are more non-western starting nodes for language & literature and religion could account for the lower non-western descendant means in the DDC, however, the permutation test keeps the number of each type of starting node constant. Descendant bias is thus not an artifact of the number of starting nodes. The mean percentage of books per starting node is roughly equal between western and non-western ones. The biggest difference is found for history in the DDC, however, the difference shows more books per non-western node than western (14.3% vs 9.1%). Thus, in the DDC, descendant bias is not fully explained by the idea that western starting nodes have more books than non-western starting nodes.

### Interim Summary

We found evidence of all three kinds of western category bias in both the LCC and the DDC. Our results also suggest that the DDC encodes a higher degree of category bias than does the LCC as the DDC has higher category count and descendant bias but the LCC has higher level bias. Of the three topics considered, religion tended to have the highest amount of bias in both systems. The amount of bias in history and language & literature varied across metrics. Language & literature did not display much count bias but displayed significant level bias in both systems, and displayed significant descendant bias in the DDC. History displayed significant descendant bias in the DDC and significant count bias in both systems. However, level bias for history was minimal, especially in the DDC where level bias favoured non-western nodes as opposed to western ones.

Our finding that both the LCC and DDC show western category bias is expected given previous work on biases in library classification (Knowlton, 2005; Zins & Santos, 2011), but our approach departs from previous work in attempting to systematically quantify the nature and extent of this bias. For example, religion is known to be a topic that shows substantial western bias (Fox, 2019), but to our knowledge previous studies have not systematically quantified the level of bias observed for religion relative to the bias observed for other topics. Similarly, there have been suggestions that the DDC shows greater western bias than does the LCC (Sultanik, 2022), but prior work has not provided comprehensive quantitative analyses to support this claim.

## STUDY 2: GENDER BIAS

We now turn from western bias to gender bias, and use our definitions of item bias to quantify gender bias at the item level in both the LCC and the DDC.

### Methods

To study item-level gender bias we worked with all books in our dataset that were classified under both the LCC and the DCC. We only considered books with non-empty author fields in their MARC records.^7^ Each of these books was tagged with the gender of its author. To determine an author’s gender, we used data from the author-name-index and author-gender tables created and kindly shared by Ekstrand and Kluver (2021) as part of their book data integration pipeline, PIReT Book Data Tools. These tables store processed versions of author name and gender data from the Virtual International Authority File (VIAF). The VIAF stores author information, including the variants of an author’s name and their gender. As discussed by Ekstrand and Kluver (2021), the VIAF, unfortunately, codes gender as binary and does not code for non-binary gender identities. Each author record is coded as either male, female, or unknown. For now, our analysis is thus limited to analyzing item-level biases between male and female authors. When more accurate author data is available, the same analyses can be performed including non-binary gender identities.

There is no linking identifier between a MARC record and its author’s VIAF record. We followed the method for linking records used in the PIReT Book Data Tools (Ekstrand & Kluver, 2021). At a high-level, string-matching was used to tag a book with its author’s gender, and there are three main cases where an author’s gender cannot be determined. The first is when a book’s author matches to a VIAF record with the gender code “unknown.” The second is when an a book’s author matches to multiple VIAF records with conflicting known gender identities and thus had an ambiguous gender code. The third is when a book’s author does not match any author record in the VIAF dataset. We discarded books in any of these three cases from our item-level analysis.

### Results

2.55 million of the 3.32 million MARC records had a non-empty author field and 1.95 million of these could be tagged with an author’s gender. Table 4 contains a breakdown of the record-matching process. Less than 1% of the records were tagged as ambiguous. Only 5% of the MARC records could not be linked to any VIAF record. These results are similar to results achieved by the PIReT Book Data Tools. In all the datasets they were applied to, somewhere between 3.3% and 6.9% of book records could not be matched to a VIAF record (Ekstrand & Kluver, 2021).

**Table 4.** Breakdown of the results of the record-matching process. No author (MARC) refers to the percentage of MARC records with an empty or missing Main Entry-Personal Name field. No author (VIAF) refers to the percentage of MARC records with a Main Entry-Personal Name field that does not match a record in the Author-name-index table.

**Subset**	**% of records**
No author (MARC)	23.12
No author (VIAF)	5.00
Unknown gender	12.36
Ambiguous gender	0.55
Known gender	58.97

#### Item Count Bias.

To measure item count bias we compared the percentage of female authors to the percentage of male authors in the books classified by the Ohio academic libraries. We found that female authors are substantially underrepresented. In the set of items with both a DDC and LCC, 16.24% of the authors are coded as female, and 83.76% are coded as male. This differs from the percentages of men and women in the VIAF dataset where 28.55% of authors with a known gender identity are coded as female and 71.45% as male. Because males and females account for roughly equal proportions of the general population, any departure from an even split provides evidence of external bias.

We also compared the circulation of books by men to the circulation of books by women. The results are shown in Table 5. The percentage of books by women in circulation is equal to the percentage of books by men. In 2007, 37% of circulating books by women were borrowed versus 29% of books by men. Similarly, borrowed books by women were taken out more times on average (3.30) than borrowed books by men (2.91). These findings reveal that demand alone cannot explain the under-representation of female authors and suggest that there are other forces that systematically reduce their representation.

**Table 5.** Circulation statistics for male versus female authors.

	F	M
% items in circulation	0.95	0.95
% items circulated (2007)	0.37	0.29
Rate of circulation (2007)	3.30	2.91

#### Level Bias.

We measured level bias by determining the most specific (deepest) category each book in the Ohio academic libraries was assigned to. We then compared the depth at which books written by women tended to be classified to the depth at which books written by men tended to be classified. In the LCC the mean depth of male and female authors is 4.55. The median for both is 4. In the DDC the mean depth of female authors is 4.65 and the mean depth of male authors is 4.45. The medians for women and men are 5 and 4 respectively.

**Figure 7.**
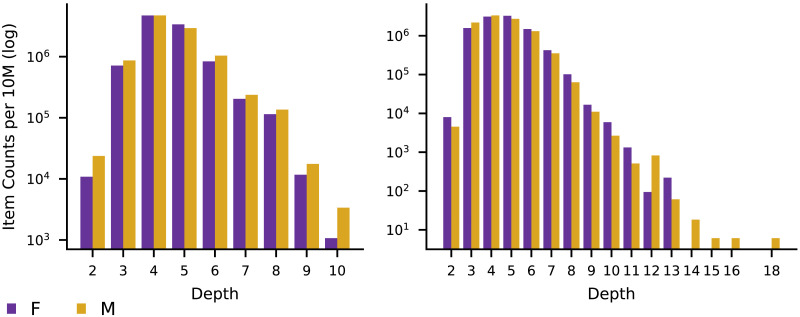
Item level gender bias in the LCC (left) and DDC (right). Data is scaled to counts per ten million.

We plotted the distributions of books over the classification tree depths in Figure 7 and computed the difference in the mean depths of books by men and women. In the LCC the difference is 0.001 and in the DDC it is 0.191. To test the significance of the results we performed a permutation test. We shuffled the classification depths among books and recomputed the difference between the mean depth of books by men and the mean depth of books by women. This was repeated 1000 times and the proportion of times the random difference in means was greater than or equal to the actual difference in means was used as the *p* value. In the LCC *p* = 0.61 and in the DDC *p* < 0.001. For the books in the Ohio academic libraries, LCC classifications did not yield a significant difference between the mean depth of classification for books written by men and the mean depth of books written by women. For the DDC the difference is statistically significant, but small.

As an alternative statistic for level bias, we calculated the probability that a randomly selected female-authored book is deeper in the tree than a randomly selected male-authored book. A male-authored and female-authored authored book were randomly sampled 10,000 times. The depths of the two books were compared to determine the number of times the female-authored one was deeper in the tree (ties were discarded). The resulting statistic measured the probability that a female-authored book would be deeper in a tree than a male-authored book, given they were not of the same depth. In the LCC this probability is 51% and in the DDC it is 57%. To test the significance of these results we performed a permutation test that measured the proportion of times the absolute value of the difference between the recalculated probability and 50% in the permuted samples is greater than the actual difference. In the LCC *p* = 0.29 and in the DDC *p* < 0.001. Again, the results do not indicate the presence of any level bias for the Ohio library books when classified using the LCC and only minimal level bias when using the DDC.

#### Distributional Bias.

Distributional bias is evident when the distribution of male authors across children of a given node tends to be flatter than the distribution of female authors. To test for this bias we collected every node that had at least 100 Ohio library books, at least 2 children, and both male and female authors. This approach yielded 822 LCC nodes and 2832 DDC nodes that could be used to compare the distributions of books by male and female authors. See Appendix C for a breakdown of the nodes that could not be used in the distribution bias analysis. To compute the distribution of male authors for each node, the number of male authors in each direct child node was divided by the total number of male authors across all child nodes. We did not use the total number of male authors in the parent node because not all items in a parent node are classified into one of the children. The same was done for the female author distribution. For example, in Figure 3B.iii there are 10 purple books and 1 is assigned to the first child node, 2 to the second, and 7 to the third. Thus the distribution of purple authors for that node is [0.1, 0.2, 0.7]. For each node, we compared the Shannon entropies of the male and female distributions to determine which of the two distributions was flatter.

**Figure 8.**
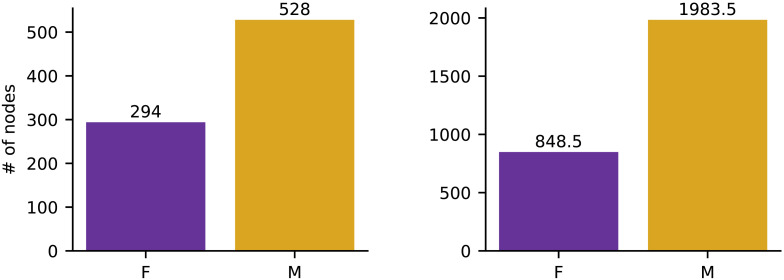
Distributional Bias in the LCC (left) and the DDC (right). The gold columns (M) show the number of nodes for which the distribution of male authors is flatter than the distribution of female authors, and the purple (F) columns show the number of nodes that yielded the opposite result. Ties were handled by adding a count of 0.5 to both columns.

Figure 8 shows the number of nodes for which the male distribution was flatter than the female distribution. In both the LCC and the DDC the distribution of male authors among a node’s children tended to be flatter than the distribution of female authors. The effect is stronger in the DDC as the relative difference in size between the two counts is 2.34 as opposed to 1.80. To test the significance of the results reported above, a permutation test was performed. The entire set of author gender labels was shuffled among the classified items. For each node, the author gender distributions were recalculated, and the same flatness comparison was applied. In both the DDC and the LCC the results were significant with *p* < 0.001.

### Interim Summary

The books in the Ohio academic libraries exhibit clear external item gender bias as there are approximately five times more books written by men than by women. When these books are classified by either the LCC or the DDC they also exhibit distributional gender bias as the books written by women tend to concentrate on a smaller subset of nodes than the books written by men. This bias is evident in both systems and stronger in the DDC. Finally, there is no strong evidence of level bias when the books are classified by either the LCC or the DDC.

## DISCUSSION

Category systems often contain inbuilt biases towards certain groups or concepts, and we developed methods that quantify these biases in hierarchical category systems. We tested our methods on two case studies of documented bias in the LCC and DDC. The first quantified western bias in the internal node (category) structure of both systems and the second quantified gender bias in the leaf nodes (items). The results confirm previous work documenting bias in the LCC and the DDC, allow for a detailed understanding of the different ways bias can manifest in the leaf nodes and internal nodes of these systems, and facilitate the comparison of bias between systems.

In the first case study, we found that language & literature in the DDC had significant category count and level bias, and that religion and history had significant count bias in both systems. These biases were in favour of western nodes and confirm previous findings that the DDC is biased in its categorization of non-western language and literature (Kua, 2008), and that non-western religions and topics are under-represented in both the DDC and LCC (Westenberg, 2022; Zins & Santos, 2011). We also found that a category system that has count bias does not necessarily have level bias or descendant bias and vice versa suggesting that the three proposed biases can be used to quantify different aspects of category bias and provide a relatively nuanced picture of how it manifests. Finally, we found that the DDC tends to show a higher degree of western category bias than does the LCC. Specifically, there was evidence of strong category count and descendant bias in the DDC whereas there was no evidence of descendant bias in the LCC.

In the second case study, we found that women are underrepresented in the set of books we considered and that there is a strong distributional bias in favour of men in both the LCC and DDC. Previous studies have documented that topics relating to women in the LCC and DDC tended to be restricted to specific categories (Olson & Ward, 1997; Rogers, 1993), and our analyses support a similar conclusion by suggesting that books by women tend to be restricted to relatively limited sections of the LCC and DDC. Despite strong evidence of item count bias and distributional bias, we do not find much evidence of item level bias in favour of men or women in either system. Like the three category biases we define, the three item biases can provide a detailed picture of the different ways in which item biases can be found in a system or the set of items it classifies.

### Why Are Library Classification Systems Biased?

Our first case study found clear evidence of western category bias, and to some extent this bias can be explained by literary warrant, or the idea that the system reflects and is justified by the literature it classifies. The system is biased because the literature is biased and it is possible that when these systems were created they were fully representative of the literature that they classified. Over time, however, library collections have changed, and although the LCC and DDC are both regularly updated, it seems likely that both embody stronger western category bias than is justified by the holdings of a modern Western university library. Some initial evidence in this direction is provided by our analysis of data from the Ohio Academic Libraries. In 2007, Ohio academic libraries held more Western than non-Western books, but we found that the relative proportion of Western and non-Western categories was not justified by the relative proportion of Western and non-Western books. In particular, we found that there was a higher average number of non-western books per non-western category than western books per western category. Similarly, although in some cases there were more western books per western starting node than non-western starting nodes, this was not consistently true. So, at least in the case of the Ohio Academic Libraries, the biases we find cannot solely be attributed to the books the system classifies.

A second possibility is that the biases reflect not the intrinsic content of the holdings, but the preferences of the users who access these holdings. For example, if library patrons were more likely to borrow Western books than non-Western books, it may make sense to have finer-grained categories for Western topics than for non-Western topics. Our analysis of circulation statistics, however, suggests that across the Ohio Academic Libraries in 2007, the average demand for each Western book was roughly equal to the average demand for each non-Western book. Differences in the preferences of modern library users can therefore not account for the finding that Western categories tend to be more fine-grained (i.e., include fewer books on average) than do non-Western categories.

Thirdly, the observed biases can be partially attributed to biased decisions made by the individuals who created these systems. Western bias has been widespread in Western culture over the past century, and has inevitably shaped the thinking of those who build and maintain library classification systems. The underlying psychological mechanisms that bias the decisions of librarians are likely to include mechanisms that drive biased categorization in general. One example is the out-group homogeneity effect, or the tendency to perceive out-group members as less diverse than in-group members. The descendant bias in the DDC seems to mirror this effect because finer-grained categories are used for Western (in-group) than for non-Western (out-group) topics. Like the out-group homogeneity effect, descendant bias in the DDC could be potentially attributed to greater familiarity with and exposure to western literature over non-western literature, or increased attention to and better memory for topics and features of literature that are relevant to the in-group (Das-Smaal, 1990; Park & Rothbart, 1982). Category count bias similarly, could also occur because features of in-group literature are easier to perceive and recall and thus it is easier to differentiate this literature and create more categories.

In our second case study, our results for gender demonstrate that the three item biases in Figure 3 are sensitive to the preferential treatment of different groups of items. As mentioned earlier, however, these biases may be the result of external social pressures affecting the items classified by a system or may be imposed on the items by the classification system itself. The item count bias we find is clearly external to both classification systems, but it is unclear to what extent the distributional bias found is internal or external to either the LCC or DDC. Comparing the two systems provides some evidence that the item biases found in the DDC have an internal component. We found that the DDC has a stronger distributional gender bias than does the LCC and had a very slight item level bias where the LCC had none. These differences occurred even though the set of books considered was held constant across the two systems. Our results therefore suggest that some proportion of item bias is internal to the DDC, but do not allow us to tell whether the LCC is also subject to internal item biases.

### Beyond Library Classification

Although our case studies focused on library classification, our methods are general and can potentially be applied to a broad range of hierarchical category systems. To illustrate, we apply our methods to WordNet (Miller, 1994). Both Western bias and gender bias are potentially relevant. For example, previous studies have documented western biases in ImageNet (Liu et al., 2021; Luccioni & Rolnick, 2023), and these biases are likely inherited from WordNet, the source of the ImageNet hierarchy. However, to illustrate the range of our methods we consider a third kind of bias. Using a procedure described in Appendix D, we identified synsets in WordNet that correspond to species of mammals, tagged these species as wild or domestic, then used our methods to measure the extent to which WordNet prioritizes domestic species ahead of wild species. Although domestic mammals account for less than 1% of all mammal species (Mammal Diversity Database, 2023), Table 6 shows that English WordNet 3.0 displays a clear bias for domestic over wild mammals. Despite a larger number of starting nodes for wild than for domestic species, count bias is present because there are more categories (i.e., WordNet synsets) overall for domestic than wild mammals. Descendant bias is also present, because domestic categories tend to have more subcategories (i.e., hyponyms) than do wild categories, leading to a more fine-grained representation.

**Table 6.** Category bias analysis for domestic versus wild mammal species in English WordNet 3.0. The 3 measures of category bias reported are the total number of synsets (Count Bias), the mean depth of starting nodes (Level Bias), and the mean number of descendants per starting node (Descendant Bias). Starting nodes are WordNet synsets corresponding to different mammal species. See Appendix D for full details of this analysis.

	**Starting Nodes**	**Count Bias**	**Level Bias**	**Descendant Bias**
**Domestic**	19	398	14.34	20.00
**Wild**	309	344	14.27	0.13

WordNet lies somewhere between an institutional category system and a natural category system, but our approach can also be used to quantify cultural and individual differences in natural category systems. Names of plants (Berlin, 1992), animals, artifacts (Rosch et al., 1976), body parts (Majid, 2010), and places (Basso, 1984; Burenhult & Levinson, 2008) are all organized into hierarchies or partonymies, and our methods could be applied to each of these cases. For example, consistent with our WordNet analysis, plant and animal names could be labelled as wild or domesticated, and future studies could measure the extent to which a folk taxonomy is biased towards domesticated ahead of wild species. The degree of bias is likely to vary across cultures in line with existing findings that agricultural societies tend to have more names for plants than do hunter-gatherer societies (Balée, 1999; Berlin, 1992; Brown, 1985). Within cultures, the degree of bias is likely to correlate with factors such as expertise (Tanaka & Taylor, 1991). For example, Aguaruna Jivaro women have much more fine-grained categories for Manioc (a tropical root crop native to South America) than do men and this difference aligns with the division of labour among men and women in Aguaruna Jivaro culture (Boster, 1985).

We defined bias as a preference for one group over another, and focused on two cases (gender and western bias) where these preferences can cause harm, especially when systems that incorporate these preferences are perceived as objective. In the case of folk taxonomy, however, a preference for domestic over wild species may be beneficial in supporting communication about the species of most interest to a given culture. Preferences are not necessarily harmful, and can instead illuminate the different needs, values, or roles of the people and cultures who create and use category systems. Our approach therefore joins a set of existing quantitative techniques that can provide insight into conceptual variation both across and within cultures (Romney et al., 1986, 2000).

### Limitations

Our work is limited in several important respects. Although our first case study revealed western bias in both the LCC and the DDC, our analysis is limited to a relatively coarse distinction between western and non-western because we are unable to determine if the actual books assigned to each category are written from a western or non-western perspective. It is likely that in a US-based library books classified as non-western still exhibit many kinds of western bias.

Our case study of gender bias was limited by its reliance on incomplete author data. As mentioned earlier, the VIAF does not account for non-binary gender identities and only labels an author as “male,” “female,” or “unknown.” In addition, for simplicity, our analyses were based on the gender of the first author or primary person associated with a book, which means that some nuance is lost because books with multiple authors of different genders are not considered.

A key limitation that applies to both our case studies is that we focused on two US-based, western classification systems. Future work could aim to apply our methods to a more diverse set of library classification systems, including non-western systems such as Russian and Chinese library classification systems (Zhang, 2003), and systems like the Universal Decimal System, which was designed to be more comprehensive than the Dewey Decimal system. It is also important to note that in both studies our book-level statistics are based on data from the Ohio Academic Libraries. The analyses of bias in the LCC and DDC based on these statistics are thus limited to how biased the LCC and DDC are with respect to this specific group of western libraries. Despite their limitations, however, our analyses seem sufficient to demonstrate that our methods are capable of capturing biases in hierarchical category systems.

### Future Work

Our work opens up several directions for future studies. We have focused on characterizing and measuring several kinds of bias, and the natural next step is to attempt to remediate these biases. For example, our methods demonstrate that the classification of non-western language and literature encodes significant level bias. The LCC has no subcategory limits so changes such as promoting the category for African languages and literatures to the subclass (second) level could help to remediate some western category bias.

Although we focused on hierarchical category systems, future work could apply some of our methods to measure bias in flat category systems. One previous study in this area focused on gerrymandering, and developed methods for quantifying bias in United States’ congressional districts (McCartan & Imai, 2023). Some of our methods for detecting category and item biases in hierarchical category systems can be directly applied to flat systems. For example, item count and category count bias can be applied without modification. Distributional bias could also be applied by considering the distribution of different groups across an entire flat system instead of considering differences in the distribution across the subcategories of each internal node.

Our work documents and quantifies biases in hierarchical classification systems, and future work could study the cognitive mechanisms that give rise to these biases. Perception, attention, and memory can all help to explain how biased collections of library books are created (Quinn, 2012), and the same three mechanisms are likely to contribute to biases in hierarchical category systems. For example, differences between in-group members are often perceived as larger than differences between out-group members, and therefore more worthy of being recognized in a classification system (Park & Rothbart, 1982). These perceptual differences may arise as a consequence of selective attention to features that are more relevant to in-group members than to out-group members (Das-Smaal, 1990). Familiarity and exposure can also lead to bias, because frequently encountered items (i.e., in-group members) are more likely to come to mind than items encountered rarely (out-group members). Laboratory experiments have previously considered all of these factors, but more work can be done to explore how these factors produce biases in hierarchical systems of categories.

Finally, our methods could be used to explore how biases in category systems change and develop with time. Category systems are rarely formed all at once and instead develop over time in response to a sequence of items. The sequence in which items are encountered can affect the categories that are created (Medin & Bettger, 1994), and future work can examine how bias is compounded or reduced as items are encountered over time. Knowlton (2005) studies historical change in the LCC by manually documenting all the ways in which the subject headings have and have not changed three decades after Berman (1971) proposed modifications to reduce bias. With access to historical versions of the LCC, DDC, or other category systems, our methods could allow us to explicitly quantify how these systems differ on measures of category and item bias with time. We expect that institutional category systems should become increasingly unbiased with time, but it is possible that some structural biases may compound and increase instead.

## CONCLUSION

We developed a suite of methods that allow us to systematically quantify and compare biases across hierarchical category systems. Although we focused on western and gender bias in library classification systems, our methods are broadly applicable and could be used to quantify cultural and individual differences in natural category systems. These methods can provide a deeper understanding of the different ways in which the needs, beliefs, and values of an individual or a group are encoded in their category systems. In cases where bias is harmful, measuring this bias can support efforts to reduce or control for this bias. *View of the World from 9th Avenue* would not score well on our definitions of category bias, but quantifying its bias could serve as a first step towards creating a more balanced categorization.

## ACKNOWLEDGMENTS

We thank Blair Armstrong, Jiangtian Li, Aida Ramezani, and several anonymous reviewers for valuable comments on an earlier version of the paper. A subset of this work was presented at the 45th Annual Meeting of the Cognitive Science Society in 2023. Katie Warburton is funded by the U of T–UoM IRTG program, and this work was also supported by NSERC Discovery Grant RGPIN-2018-05872 and by ARC Future Fellowship FT19010020.

## AUTHOR CONTRIBUTIONS

K.W.: Conceptualization; Data curation; Formal analysis; Methodology; Software; Validation, Visualization; Writing – original draft. C.K.: Conceptualization; Funding acquisition; Methodology; Supervision; Writing – review & editing. Y.X.: Conceptualization; Funding acquisition; Methodology; Supervision; Writing – review & editing. L.F.: Conceptualization; Funding acquisition; Methodology; Supervision; Writing – review & editing.

## DATA AVAILABILITY STATEMENT

The code and processed data are available on OSF: https://osf.io/wd9c6/.

## Notes

^1^ Code for this project can be found at https://osf.io/wd9c6/.^2^ See *View of the World from 9th Avenue*.^3^ This work contains information from OhioLINK Circulation Data which is made available by OCLC Online Computer Library Center, Inc. and OhioLINK under the ODC Attribution License.^4^ The MARC (machine-readable cataloging) 21 Format for bibliographic data is a digital format used to describe items catalogued by libraries.^5^ A list of category tags can be found in the linked code repository.^6^ In the LCC 571 of the categories were from the topic Religion, 920 from Language & Literature, and 1518 from History. In the DDC 2420, 7758, and 3358 categories were from Religion, Language & Literature, and History respectively.^7^ Author names are stored in the Main Entry-Personal Name field of a book’s MARC record. This field records the person mainly responsible for the work (Library of Congress, 2022), whether they are the primary author in a multi-authored work or the editor of an anthology. For simplicity, we use the term “author” in all cases.^8^ All copyright rights in the Dewey Decimal Classification system are owned by OCLC, Inc. Dewey, Dewey Decimal Classification, and OCLC are registered trademarks of OCLC, Inc.^9^ Sometimes a Wikipedia name directly linked to a BabelNet ID (e.g., “Bali_cattle”) and other times it had to be converted to lowercase (e.g., “dog”). We therefore considered both possibilities before concluding that a BabelNet entry for a Wikipedia page could not be found.^10^ Again, some synsets have multiple parents (hypernyms). If this was the case, we took the average depth between the parents and counted that as the starting node’s depth.

## APPENDIX A: LCC AND DDC CATEGORY EXAMPLES

Subcategories of the category “Handicrafts. Arts and Crafts” in the LCC and its DDC equivalent “Handicrafts” (Table A1).

**Table A1.** “Handicrafts. Arts and Crafts” (TT1-999) subcategory names in the LCC and “Handicrafts” (745.5) subcategory names in the DDC.

**LCC**		**DDC**	
**Label**	**Name**	**Label**	**Name**
TT161-170.7	Manual Training. School shops	745.51	Wood crafts
TT174-176	Articles for children	745.52	Fiber crafts
TT180-200	Woodworking. Furniture Making. Upholstering	745.53	Leathercraft
TT201-203	Lathework. Turning	745.54	Paper crafts
TT205-267	Metalworking	745.55	Shellcraft
TT300-382.8	Painting. Wood Finishing	745.56	Metal craft
TT387-410	Soft home furnishings	745.57	Working with polymer clay
TT490-695	Clothing manufacture. Dressmaking. Tailoring	745.58	Working with beads
TT697-927	Home arts. Homecrafts	745.59	Making toys
TT950-979	Hairdressing. Beauty culture. Barbers’ work		
TT980-999	Laundry work		

## APPENDIX B: COMPUTATIONAL DETAILS

To create a tree structure representation of the LCC we parsed Excel files representing the 21 top-level classes of the LCC. The Excel files were created by extracting data from the 21 LCC outlines stored at https://www.loc.gov/catdir/cpso/lcco/. In the outlines, tabbing is used to indicate the hierarchical relationships between categories. In the Excel spreadsheets rows and columns were used to represent this relationship. For example, consider two categories where category A is stored in the first row of the spreadsheet and category B in the second. If category A is in the first column and category B in the second then category B is a subcategory of A. If B is also in the first column then A and B are at the same classification depth.

To create a tree structure representation of the DDC we used two sources. The first was a set of Excel versions of the OCLC DDC 22 summaries^8^ at https://www.oclc.org/research/activities/browser/terms.html. These summaries represented the first 3 levels of categorization in the DDC. For categories at depths greater than 3, we used the MDS table at https://www.librarything.com/mds/. Only categories associated with both a name and a classification number were used. In the DDC the hierarchical structure of the system is represented by the position of the digit that differentiates a category. After the third level, digits occur following a decimal place. For example 295.5 is a subcategory of 295.

Finally, to represent the books (or leaf nodes) in the LCC and DDC we parsed MARC records using the Pymarc library. MARC records contain noisy data, so string processing was used to create a uniform format for LCC and DDC numbers respectively. For the DDC this meant that the number had to be at least 1 digit long, contain exactly one decimal if greater than 3 digits, and contain only digits otherwise. For the LCC this meant that the number had to be at least two characters long, contain at least one alphabetic character, and contain at most one number that falls within the range of 1 to 9999. The number can be a natural number or a decimal number and the alphabetic characters have to precede the numeric ones. Both DDC and LCC classification numbers can contain Cutter numbers. These are additional alphanumeric character sequences that follow another decimal (Cutter). These numbers provide information about a book such as author, publication date, and additional subject information. Cutter numbers were discarded as they were not related to the general category structure of the system. If a book’s classification numbers could not be converted to the regularized format the book was excluded from analysis. Next, for each record with both a DDC and LCC, we determined if the LCC and DDC numbers were valid classification numbers in their respective systems. We called a classification number valid if it could be placed into a category in our tree structure representations. To place books into categories we found the deepest category that its classification number fell into, and then recursively added that book to the parents of the category.

## APPENDIX C: DISTRIBUTIONAL BIAS NODE DATA

Table C1 describes the nodes that were excluded from the distributional bias analysis because they either had fewer than 2 child nodes, fewer than 100 books classified under them, or did not have books written by both men and women.

**Table C1.** Counts of nodes in the LCC and the DDC that did not meet the criteria for use in the distributional bias analysis because they had fewer than 2 children, fewer than 100 books, had books written by men but not women, and vice versa. The column named ‘Valid nodes’ reports the counts for nodes that could be used in the analysis.

	< 2 children	< 100 books	No women	No men	Valid nodes	Total
LCC	5858	3816	1116	50	822	6870
DDC	27,668	28,120	8167	824	2832	33,751

## APPENDIX D: DOMESTIC BIAS IN WORDNET

To study bias in WordNet we first extracted a list of 5587 unique Wikipedia page names, each corresponding to a page for a different mammal species. 28 of the names represented pages for domesticated mammal species and 5559 were for wild species. The names were scraped from several Wikipedia pages (Table D1) that contained lists of various mammal subgroups. The domestic mammal names were taken from a page that listed all domesticated animals, and we manually collected names from this page to ensure that we only had names for mammals. The domesticated animals list sometimes contained subspecies that were domesticated variants of a species that had both wild and domestic subspecies. To keep the analysis consistent we used the mammal species as opposed to the subspecies and counted it as domesticated. As of November 2023, the Catalogue of Life checklist (Bánki et al., 2023) lists 5952 extant species of mammals, so our collected list of mammal species seemed relatively comprehensive. Any mammal species that does not have a Wikipedia page is unlikely to have a WordNet synset. We therefore believe that the domestic and wild mammal lists collected from Wikipedia provide a sufficient basis for collecting synsets for these species in WordNet.

**Table D1.** Wikipedia pages that were scraped to get Wikipedia IDs for pages corresponding to different mammal species. The indented pages were linked on “List of placental mammals” and scraped individually.

**Page**	**URL**	**# of species**
List of domesticated animals	https://en.wikipedia.org/wiki/List_of_domesticated_animals	28
List of placental mammals	https://en.wikipedia.org/wiki/List_of_placental_mammals	232
List of rodents	https://en.wikipedia.org/wiki/List_of_rodents	2495
List of primates	https://en.wikipedia.org/wiki/List_of_primates	531
List of lagomorphs	https://en.wikipedia.org/wiki/List_of_lagomorphs	89
List of bats	https://en.wikipedia.org/wiki/List_of_bats	1246
List of carnivorans	https://en.wikipedia.org/wiki/List_of_carnivorans	293
List of artiodactyls	https://en.wikipedia.org/wiki/List_of_artiodactyls	348
List of monotremes and marsupials	https://en.wikipedia.org/wiki/List_of_monotremes_and_marsupials	349

To link Wikipedia pages for mammal species with WordNet we used BabelNet (Navigli & Ponzetto, 2012), a multilingual semantic network that links Wikipedia pages to WordNet synsets when the same concept exists in both datasets. Thus, using the lists of Wikipedia page names for wild and domestic mammal species, we queried BabelNet to see whether a BabelNet entry for each page existed, and if so whether this entry contained a WordNet ID. If a WordNet ID existed then we extracted the WordNet synset corresponding to the queried mammal species.^9^ All of the Wikipedia pages for domesticated mammals linked to BabelNet entries, and 165 (2.97%) of the wild mammal Wikipedia pages were not found in BabelNet. Of the 28 domesticated BabelNet entries 19 (67.86%) were linked to unique WordNet synsets. Of the 5394 wild BabelNet entries, 309 (5.73%) appeared in WordNet. We treated the synsets corresponding to mammal species as starting nodes and did not analyze any categories that were parents (hypernyms) of them. We therefore worked with 19 domestic starting nodes and 309 wild starting nodes.

WordNet is not a strict hierarchy and sometimes synsets can be descendants of multiple nodes. To measure count bias we therefore counted all unique synsets that either belonged to the list of starting nodes or were descendants of these starting nodes. There were more domestic nodes than wild nodes in WordNet (398 and 344 unique synsets for domestic and wild mammals respectively). To measure level bias we calculated the mean depth across all domestic and wild starting nodes individually. The depth was measured from the synset, “entity.n.01”, at the top of the WordNet hierarchy.^10^ Domestic starting nodes, on average, were slightly deeper in the WordNet hierarchy than wild starting nodes (average depths were 14.34 and 14.27 for domestic and wild nodes respectively). Finally, to measure descendant bias we calculated the mean number of descendants across domestic and wild starting nodes. We counted all direct and indirect hyponyms below a synset as descendants. Domestic starting nodes had, on average, 20 descendants whereas wild starting nodes had 0.13. The low result for wild nodes indicates that most of these nodes had no children. The results for all 3 category biases are summarized in Table 6.

The WordNet analyses just described were carried out at the category level (Figure 2) rather than the item level (Figure 3). For any given person, the items in their semantic representation would be individual mammals they had encountered (e.g., their dog Rover). WordNet has instances of some categories (e.g., Barack Obama is an instance of President of the United States) but not for animals, and is therefore best suited for category-level rather than item-level analyses.
